# A Review of Current Challenges and Case Study toward Optimizing Micro-Computed X-Ray Tomography of Carbon Fabric Composites

**DOI:** 10.3390/ma13163606

**Published:** 2020-08-14

**Authors:** Armin Rashidi, Tina Olfatbakhsh, Bryn Crawford, Abbas S. Milani

**Affiliations:** Composites Research Network-Okanagan Laboratory, School of Engineering, University of British Columbia, Kelowna, BC V1V 1V7, Canada; armin.rashidi@ubc.ca (A.R.); tolfat@mail.ubc.ca (T.O.); bryn.crawford@ubc.ca (B.C.)

**Keywords:** fabric composites, micro-computed X-ray tomography, image processing

## Abstract

X-ray computed tomography provides qualitative and quantitative structural and compositional information for a broad range of materials. Yet, its contribution to the field of advanced composites such as carbon fiber reinforced polymers is still limited by factors such as low imaging contrast, due to scarce X-ray attenuation features. This article, through a review of the state of the art, followed by an example case study on Micro-computed tomography (CT) analysis of low X-ray absorptive dry and prepreg carbon woven fabric composites, aims to highlight and address some challenges as well as best practices on performing scans that can capture key features of the material. In the case study, utilizing an Xradia Micro-CT-400, important aspects such as obtaining sufficient contrast, an examination of thin samples, sample size/resolution issues, and image-based modeling are discussed. The outcome of an optimized workflow in Micro-CT of composite fabrics can assist in further research efforts such as the generation of surface or volume meshes for the numerical modeling of underlying deformation mechanisms during their manufacturing processes.

## 1. Introduction

Micro-computed tomography (Micro-CT) is becoming increasingly important among the non-destructive inspection (NDI) techniques available for industrial applications, where the three-dimensional (3D) nature of the material is important, or where the evolution of critical features is of interest, either during manufacturing or in-service conditions [1]. In the field of polymer composites, these are both key attributes: the inherent heterogeneity and architecture of fiber-reinforced composite structure often require 3D, multi-scale assessments, while understanding the nucleation and evolution of defects is critical to the overall structural integrity of the composite [2]. Unique insights can be obtained from Micro-CT, regarding processing or in-service degradation. At the same time, different types of Micro-CT scanners can offer detailed information across the length scales, from the whole part to laminates sections, and from yarns to the individual filament level [3].

For polymer composites, qualitative 3D imaging makes it easy for designers to map and assess the presence of manufacturing defects, or to identify the occurrence of specific damage modes. In general, a great deal of information can be obtained by scanning through 2D radiographs or segmented and rendered 3D images to locate features of interest. Nonetheless, voids, matrix cracks, fiber breakage, delamination, and their interconnectivity should be visualized in 3D, overcoming the potential misrepresentation from 2D characterizations. In order to model the mechanical properties and failure of composite structures, it is essential to determine the tow architecture, and particularly, any local deformations due to the interaction of adjacent tows during compaction/consolidation [4], where in-situ tow visualization has been accomplished in the past by a labor-intensive serial sectioning technique [5]. Images of the specimen edge are obtained utilizing an optical or scanning electron microscope (SEM) by progressively removing small depth of edge material which allows the capture of a series of sectional micrographs. These images can then be reconstructed to display the yarn architectures. Micro-computed tomography, however, is an alternative method for this process. It has the benefit of being non-destructive and allows a large amount of the internal geometric features to be captured with a single scan. Internal cross-sections are also visualized, which in turn allows the reconstruction of three-dimensional volumes.

Although recent decades have seen a sharp increase in the capability of Micro-CT, in particular, laboratory-based tomography and its application to the multi-scale studies of composites, challenges remain. In the case of laboratory Micro-CT, the volume scanned is often fairly small compared to the composite architectures and components. Conversely, Synchrotron X-ray imaging allows larger components to be studied, in addition to the fact that images of composites can suffer from poor phase contrast and long acquisition times [6]. Direct use of industrial Micro-CT devices may not provide the expected results, due to the high complexity of the samples with geometrical details at different length-scales. Therefore, efficient post-processing techniques are required to achieve a high-quality assessment of the microstructure [7]. Moreover, a variety of parameters could prohibit the operator from conducting an accurate scan. Sample preparation, handling, positioning, and alignment play a significant role in the final quality of the reconstructed projections. Fibrous composite materials such as woven fabrics are more prone to these unintended in-plane or out-of-plane translations and therefore need to be immobilized during the scan [8].

### 1.1. General Challenges in 3D Imaging of Advanced Composites

In the absorption-based imaging of Micro-CT scanning processes, contrast is generated based upon differences in X-ray attenuation of materials, which is mainly associated with density [9]. Internal cross-sectional visualizations and three-dimensional models of the sample can then be generated from the point cloud data. According to Equation (1), linear attenuation coefficients are used as a source of contrast on carbon composites:

(1) $$\mu =ln\left(I_{0}/l_{x}\right)/x$$

where $l_{x}$ is the intensity at a depth of x cm, $I_{0}$ is the original intensity, and μ is the linear attenuation coefficient, it is difficult to discriminate between fiber and resin interfaces, due to the infused fibers and resin having similar densities and atomic compositions [10,11] (e.g., linear attenuation values of 0.354 vs. 0.342 at 40 keV, respectively). It is even more challenging to differentiate between fibers at contacting interfaces, particularly if the fibers in the respective tows are aligned in the same direction and acquisition artifacts are present. Moreover, at some spatial locations, the linear attenuation coefficient will contain averaged information from fibers and resin. Hence, there exists a need for contrast enhancement as well as careful selection of voxel size. Material properties of carbon fiber reinforced polymers (CFRPs) differ according to their orientation; namely, physical properties along fibers and perpendicular to fibers are significantly different. Moreover, one of the main goals of Micro-CT analysis on fibrous composite structures is to track the orientation of the fibers along the length and through the thickness to investigate the effect of process parameters on fiber misalignment [12], which is achievable with sufficient contrast. Therefore, fiber orientation must be inferred from X-ray images and the actual fiber orientation must be considered, particularly when generating finite element models to address the effect of fiber misalignment on part performance. Comparatively, for glass fiber-reinforced plastics (GFRPs), the successful generation of a three-dimensional finite element model that reflects the actual fiber orientation has been reported in previous studies [13]. This is attributed to a larger diameter size (~21 microns) and sufficient difference in density of glass and epoxy resin. However, as mentioned, it is problematic to distinguish fibers from resin for CFRP clearly on Micro-CT images. The propagation-based imaging (also called in-line phase contrast) technique has been shown to effectively capture the discontinuities between different phases by enhancing the phase contrast as the sample to detector distance (propagation distance) increases towards the Fresnel regime, creating bright fringes around discontinuities [1], as opposed to the conventional absorption contrast method. A comparison between the contrast-enhancing methods will be discussed in Section 2.2.3. Contrast agents with a high atomic number, e.g., zinc iodide can also be used to improve the absorption contrast [14].

The sample size and view window resolution are yet more factors affecting the quality of reconstructed two-dimensional (2D) projections. The spatial resolution affects the level of detail that can be distinguished from an image. On the one hand, the resolution needs to be selected on the basis of length-scale, with respect to the features under investigation. Given that the feature resolution is generally around two to three times the pixel size [15], this places a lower limit on the voxel size that can be chosen. On the other hand, conventional reconstruction algorithms require that the object is entirely within the field of view, more specifically in the through-thickness direction. This represents one of the main limitations when using the current generation of Micro-CT systems to study advanced composites. If individual fibers must be imaged (carbon fibers are typically 7–8 µm in diameter) then the sample thickness must be a few mm in size, given that the observed composite behavior is often inherently affected by the sample size [16]. The use of small samples may be justified when damage mechanisms are studied, or when qualitative and quantitative analyses are required to inform and validate models. However, whether the results obtained on smaller scale composite samples can be translated to larger length-scales needs to be assessed in each case. In addition, some fabric architectures, such as woven composites, have repeating patterns that spread over significant distances, meaning that the representative volume element (RVE) may be larger than what can be imaged at an acceptable resolution which guarantees distinction between fibers and the matrix at their interface [17].

Various strategies can be employed to overcome the sample size to resolve issues. Stitching multiple images together to form an enlarged composite image can be used to extend the field of view illuminated using a fine pixel size [18]. When stitching is not adequate to capture the whole width of the image, a single region of interest (ROI) might be used [19]. The key benefit of this approach is the capability to screen a sample at a coarse scale and then to identify an ROI for subsequent high-resolution imaging, without having to cut the sample physically.

Extracting quantitative information from Micro-CT images requires appropriate image processing (e.g., filtering) and further segmentation. Segmentation is the process by which voxels in the 3D image are assigned into groups, either manually or increasingly computationally, often based on their grayscale values [20]. Segmentation is a challenging task mostly due to (1) wide imaging variability resulting from different imaging modalities, scanning protocols, and scanners parameters; (2) large intra- and inter-sample structural variability; (3) intensity value overlap between a structure’s body and its proximity [21]. Quantification of the morphology, distribution, connectivity and volume fraction and the segmented phases can then be undertaken. It is possible that errors are introduced during segmentation [6]. These errors include the quantity (number of segmented objects), the area of the segmented objects, the contour (degree of boundary match), and the content (existence of inside holes and boundary holes in the segmented region) [22,23]. They may arise from artefacts introduced during acquisition, the convergence of automatic thresholding algorithms and/or they can be user-induced in the case of manual segmentation. It may also miss key aspects that can be picked up by inspecting sequences of slices by eye and therefore, segmentation often requires manual intervention. Segmentation made by automatic thresholding works well in low noise projections with sufficient contrast between different phases [24]. In cases where the features are less distinct, or where noise or artefacts (such as wrongfully pronounced edges in phase-contrast CT) from scanning introduces significant greyscale variations, more sophisticated segmentation algorithms [25] may be utilized [26]. When manual adjustments are required, the subjectivity of the user needs to be taken into account. Regarding composites, quantitative Micro-CT analysis can be used to identify the optimum processing conditions by quantifying manufacturing defects (e.g., void volume fraction [24], fiber misalignment and wrinkling [27], etc.). Micro-CT quantifications can also be used to assess the extent of damage (e.g., matrix cracks or fiber breakages [28], the area and volume of delamination [29]) in relation to the loading scenario or service life, or to follow composite degradation as a function of loading conditions and environment.

Fiber-scale and tow-scale segmentation and skeletonization based on Micro-CT images have attracted attention recently [30]. Classical methods consist of thresholding, region-based, and edge-based methods. In thresholding, partitioning is done by thresholding the image intensity; pixels with intensity higher than the threshold belong to class “A” and the ones less than the threshold are in class “B” [31]. In more complicated thresholding methods, the image is first divided into sub-images and then simple thresholding is applied to each sub-image. The second type of classical segmentation, region-based method, aims to group pixels with similar intensities. Watershed method [32] is one of the most important algorithms of this type, which is used widely in the image processing software. This method analogously translates the pixel’s intensity as the height of the regions in a landscape flooded by water and tries to find the basins and ridgelines of the watershed. The other classical segmentation method is finding edges in the image based on the local gradient of the image intensity. In the edge-based methods, changes in the intensity of the image in a region indicate a high chance of the existence of an edge in that region. There are different edge detector operators applied to such methods, including Prewitt, Sobel [33], Roberts [34] and Laplacian of Gaussian operators [35]. Pattern recognition-based methods [36] use pixel classification and include unsupervised clustering, and supervised classification. The former groups a set of objects based on their similarity within a feature space, and the latter categorizes examples in a labeled training set to create a model that can classify future and unlabeled examples. Deformable models iteratively move the shape to find the best configuration based on the minimum total energy. Wavelets-based methods [37] are statistical algorithms in which a set of parameters from the wavelet coefficients of any pixel of the image is picked, and the distance between these parameters and the parameters of different regions of the image are compared, to find the best region that matches the pixel. Atlas-based and knowledge-based segmentation methods [38] are mostly used in medical imaging and are not covered in this study. The development of automated segmentation procedures able to segment individual carbon fibers is technically problematic, because of their low contrast with polymer matrix, and small fiber diameter. It is further complicated by high fiber volume fractions, where fibers are densely packed. However, algorithms specifically designed to extract tightly packed fiber paths are now being established [27].

### 1.2. State of the Art in Micro-CT of Woven Fabric Composites

Fabric reinforced composites are often composed of internal microstructure defects, including fiber misalignment/wrinkling, porosity, and resin-rich regions. These defects have various causes such as thermal residual stress, [15] out-of-plane stitching, [39] or poor resin impregnation during consolidation [40]. Critical assessments of the internal geometry after the manufacturing are vital for developing more reliable and robust production methodologies.

Studies have been conducted previously, e.g., on woven glass-fiber composites to investigate dimensional variability of tow architecture [41], and to generate three-dimensional visualizations [42]. Additionally, X-ray microtomography has been used to investigate micro cracking in carbon-fiber and glass-fiber composites [14], and for determining material composition based on density differences [43]. Similarly, micro-CT has been utilized to investigate the internal structure of other hybrid composite materials in the literature such as woven composite laminates with carbon nanotubes [44], chopped carbon-reinforced tapes [45], chopped fiber reinforced foams [46], short carbon-reinforced ABS (acrylonitrile butadiene styrene) preforms [3], uncompressed relaxed fibers [41], dentin–dentin bonding systems and composite systems [15] and stitched carbon epoxy composites [16]. The permeability and impregnation of the fiber reinforcements were quantified using the Micro-CT for a non-crimp carbon textile in [17] and an out-of-autoclave carbon/toughened epoxy prepreg in [18]. A sheared geometry at the unit cell level was investigated in [19] for a single layer E-glass non-crimp 3D weave. The manufacturing-induced voids (arising from air entrapment during resin flow, gas created because of chemical reactions during the curing process, and nucleation of dissolved gases in the resin [47]) were quantified for unidirectional and fabric carbon epoxy composites [48], multi-axial lay-up of unidirectional carbon weave [49], and carbon fiber reinforced SiC-matrix composites [50]. The use of Micro-CT has also been employed to characterize the damage state during the crack opening for toughened carbon fiber reinforced epoxy [51]. Fatigue damage evolution was studied by Cosmi and Bernasconi for a short glass fiber-reinforced Polyamide resin PA6 [52] and a unidirectional non-crimp fabric reinforced epoxy using the Micro-CT [53]. In addition, the structural damage due to UV light was investigated for different epoxy resin composites [54]. Baran et al. [55] investigated the internal geometry of glass/polyester pultruded profiles using Micro-CT. Poor alignment of the fibers and resin-rich areas were successfully captured, and porosity was characterized, using density segmentation.

With the developments in digital image acquisition and image processing techniques, the quantification of structural features, 3D model rebuilding together with the finite element (FE) mesh generation is considered to be a significant trend of current Micro-CT research. The combination of the reconstructed 3D model with the FE mesh generation and numerical analysis has been reported by several research groups [56]. A comparison was presented between experiments, with simulations obtained from Micro-CT and simulation based on an idealized geometry in the case of a transverse compression test of a carbon twill reinforcement [57]. In the manufacturing process of composite parts, the forming behavior of textile reinforcements profoundly influences the final quality of the component. The ability of the fabric to form into different shapes is primarily related to the fabric architecture, which affects the impregnation and mechanical properties of the final composite part [58]. The forming of fabrics induces shear deformation (simple shear, i.e., shear linked to the rotation of yarns in intersections, and shear slip, i.e., shear linked to rotational movement of yarns in intersections [59]), which alters local fiber volume fractions and the geometrical structure of the reinforcements. These changes can affect the permeability of the fabrics and will influence the impregnation process and, finally, the composite’s mechanical properties. Many of these local features can be quantified to inform computer models through well-executed micro-CT scans entailing the accurate geometrical properties, thickness variations, fiber orientation (i.e., anisotropy directions of the material) and the fiber volume fraction. It has been shown that that the description of the variability considered by the Micro-CT based FE models, compared to geometrical modelers, yields better results [60] (see also Section 3.2.2).

## 2. Case Study

To further address and visualize some of the main challenges reviewed above, a case study is presented next, where scans were performed and compared on both dry and prepreg carbon-fiber fabrics with a plain weave pattern. Further critical view of the art of Micro-CT practice for polymer woven composites is provided throughout the example, including some general considerations to improve the related data acquisition and analysis. A short comparative analysis with X-ray “contrast enhancement” techniques has also been made. Finally, filtering, segmentation and meshing techniques are discussed, followed by results of cross-sectional and three-dimensional reconstructions.

It should be noted that Micro-CT is known to be a highly complex process, which requires the user to optimize a decision space with many physical, optical and geometrical factors that interact non-linearly with higher-order effects. As such, some steps in this process are heuristic in nature and are in part a function of the type of sample being used, the available equipment, software employed and therefore, cannot be easily generalized or parameterized. The process can hence become highly iterative at particular steps, leading to high time investments and experiential learning. In light of these realities, a contributing intent of the present case study is to provide better insight and considerations that can ultimately reduce the time needed for optimizing the Micro-CT process of CFRPs, particularly for non-experienced users.

### 2.1. Materials

**Figure 1:**
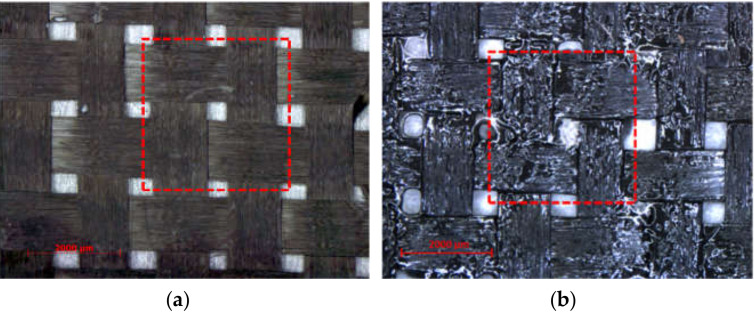
Materials used for the scanned samples (**a**) 3 K plain weave dry fabric; (**b**) Carbon/Epoxy prepreg with the same architecture as (**a**). The scanned region of interest (ROI) is shown in the red box.

The materials used in this study were a dry carbon fabric with a plain weave pattern (widely used in the fabrication of composite parts) and a Carbon/Epoxy prepreg provided by Cytec Industries (Woodland Park, CO, USA). As shown in Figure 1, the fiber tows in both warp and weft were 3 K and equally spaced in both directions. The dimension of both specimens was 6 by 10 mm, however, a unit cell with a size of 4 by 4 mm was scanned and reconstructed to mimic the repetitive pattern of the composite fabric [61]. Both materials share the same weave architecture, spacing and filament counts in warp and weft directions. The characteristics of the test fabrics are provided in Table 1.

### 2.2. Micro-CT Acquisition

#### 2.2.1. Principles

**Figure 2:**
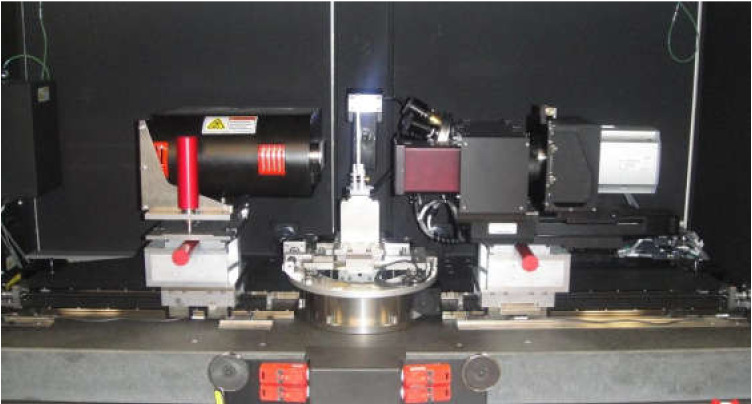
The Xradia X-400 Micro-CT industrial imaging suite used in the case study. This setup contains an X-ray source (left component), the stage for positioning the sample and region of interest (center) and an X-ray detector for obtaining transmitted photons that have passed through the sample (right component). The X-ray detector features a turret with multiple objectives at different magnifications. Namely, 4 Megapixel (2048 × 2048) 16-bit digital CCD camera (Andor DW436-BV-550) incorporated five microscope objectives (0.39×, 1×, 40×, 10× and 20×) with scintillators.

Micro-computed X-ray tomography is the process of imaging samples, using x-ray wavelength photons. As such, it is critical for users to be cognizant of the nature and properties of the x-ray beam being used in Micro-CT, as it is a major driver in determining the outcomes of the imaging process [9]. In this study, an Xradia X-400 Micro-CT (Carl Zeiss X-ray Microscopy, Inc., Pleasanton, CA, USA) suite was used for the imaging of composite woven fabric. This device uses a cone-beam arrangement to image samples. The system configuration is illustrated in Figure 2. A cone beam-based system is primarily composed of a local x-ray source, a stage for holding the sample of interest that allows for the transmission of photons through the sample, as well as a detector for registering the energy and the number of photons that strike a sensor array, having passed through the sample [62]. The two main source parameters that can be adjusted, with respect to designing imaging experiments, are the energy of the photons (controlled by varying the applied voltage), as well as the total number of photons (controlled by changing the current or exposure time). The energy of the photons exiting the source follows a probabilistic distribution, hence this parameter is not deterministic in nature, yet the variance of the energy can be limited through high-tolerance system design and the average of the distribution can be controlled through direct means [63]. The probabilistic nature of the x-ray beam demonstrates the need for employing best practices, as a means for limiting the effect of noise on the Micro-CT imaging results.

There are many other choices of parameters that users must deliberate on, in order to obtain a high-quality image. As Micro-CT is concerned with imaging, the laws of optics govern much of the process. Hence, the distance between the sample-to-source, as well as the sample-to-detector, play important roles in defining the view window sizes, geometrical magnification, image quality, and other important factors [64]. Moreover, the material(s) incident to the x-ray beam have different attenuation properties on the beam and thus, choosing these parameters with the material in mind is critical. In order to obtain sufficient information to resolve internal details about the sample and perform 3D reconstruction, many different projections are required (e.g., 2500 projections were used for all scans in the present study). The greater the number of projections, the more detail can be resolved from the sample of interest [62].

Once the projection images are captured, software techniques such as iterative reconstruction (IR) with statistical modelling, back projection, and filtered back projection (convolution method, also used in this study) [65,66] can be employed to reconstruct the scanned volume. In order to perform this activity, complex mathematical operations must be performed, requiring a transformation of the system into polar coordinates, calculating the Fourier transform for each measured projection at each respective angle, convoluting the function with another weighting function and then finding the inverse Fourier transform, in order to generate a set of frequency components that allow for the determination of internal material and geometric features. The fundamental concepts of Micro-CT imaging demonstrate that throughout the process, there are many approximations made when capturing the physical internal structure of a sample into a series of reconstructed digital images. The need for best practices, particularly when imaging noisy materials, is necessary for generating highly precise and accurate images for use in further modeling activities.

#### 2.2.2. Optimizing Sample Mounting, Scan Parameters and Filter Selection

Resolution and voxel size are both dependent on magnification, desired FOV (field of view), and distance of the sample from the x-ray source and the detector [67]. If both a large field of view and small voxel size are required, several overlapping scans with small FOV need to be captured to cover the desired large FOV [53]. In the present work, capturing one unit cell of a woven fabric composite was of interest. To this end, the source and detector were moved and the magnification was adjusted, in order to optimize the resolution for the fixed FOV of a unit cell. However, the sample size is bigger than one unit cell to prevent fabric architecture from breaking apart during sample preparation.

**Figure 3:**
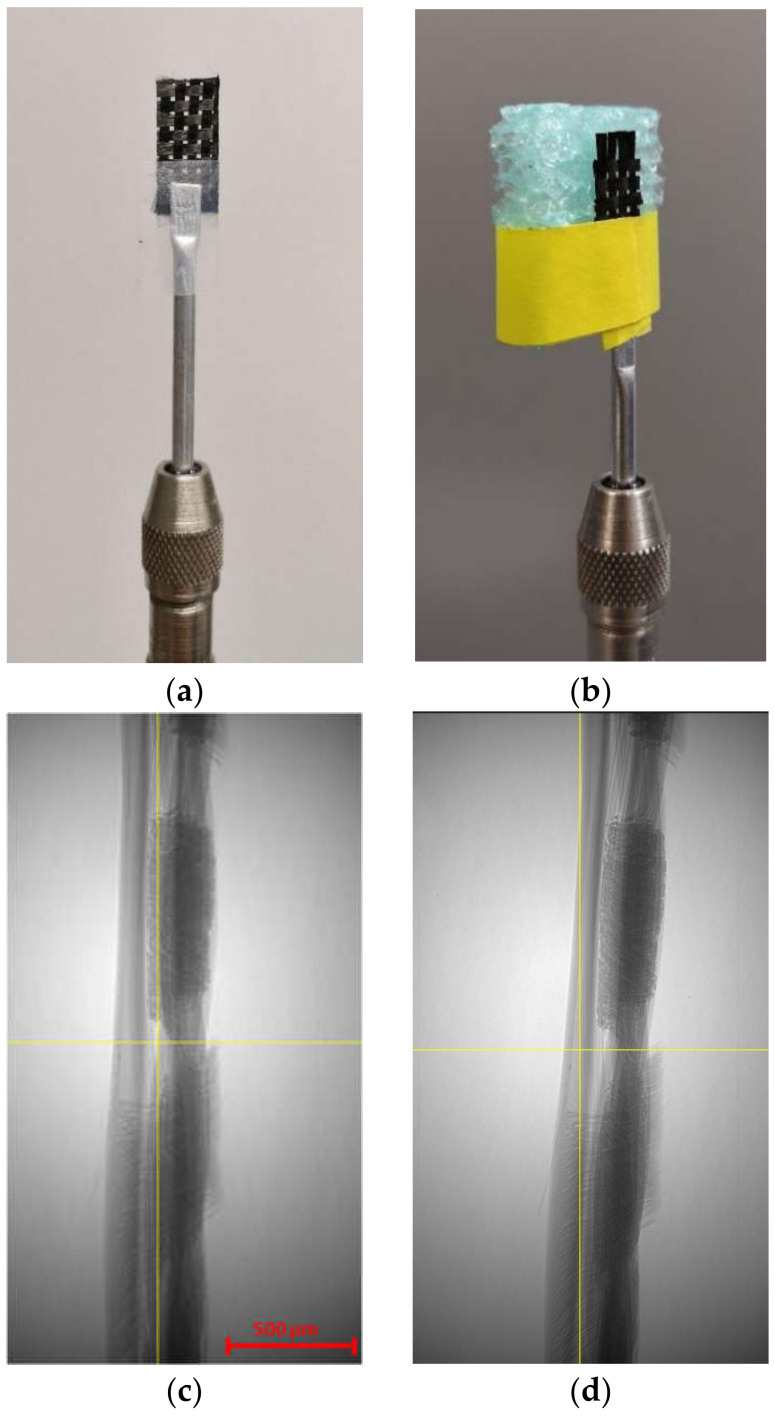
(**a**) The first trial of fixing the carbon dry fabric on the holder; (**b**) properly mounted sample to avoid wobbling during the scan; (**c**) position of the sample before scan after the first trial; (**d**) position of the sample after scan without the foam holder (after 5 h).

One of the challenges for imaging woven fabric composites is fixing the sample, since any vibration or sample wobble during the scan may result in a blurred 3D image. Although the alignment with the axis of rotation can be adjusted by converging the center shifts during the reconstruction step, a wobble of more than 3–5 degrees causes deviation of the center shift along the height of the sample and therefore results in blurry images with no clear interface between the filaments. The low bending stiffness of the dry woven fabric and rolling the weave for packaging and transportation makes it prone to move and curl or unfurl, during the rotation of the fixture. In the present study, the sample was first attached to the holder by transparent tape, as shown in Figure 3a. The holder was then placed into a rotational fixture, which is mounted inside the Micro-CT suite. However, by comparing the 2D radiograph before and after the five-hour scan, a significant wobble was observed (~5 degrees). Figure 3c and d illustrate the position of the sample with respect to the centerline before and after the scan, respectively. A new mounting routine depicted in Figure 3b was proposed in order to minimize the wobbling and sample movement during the scans (see Section 2.2.4).

After mounting the sample, the optimum values for the scan parameters must be found to yield a sufficient intensity contrast between the different constituent materials in the object, and between the objects and background space. The high intensity of the emitted X-ray beam is the result of the high voltage and large current applied to the tube. The optimal energy, proportional to the square of the voltage, depends on various factors, such as material type, object thickness, and the background space in which the material is scanned [68]. High voltages provide enough intensity for high-density materials. On the other hand, low voltages enhance the contrast but require a larger image acquisition time and may not work for dense materials with greater X-ray attenuation. Accordingly, there is a trade-off between contrast and intensity, while selecting the optimal voltage [69]. As previously stated, increasing the tube current also increases the X-ray intensity, but does not change the distribution of X-ray energy. However, increased electron emission results in the spreading of the X-ray focal spot and consequently reducing the resolution (i.e., increasing penumbral blurring) [62]. The voltage range of the Xradia MicroXCT-400 suite is 40-150 kV and the maximum power is 10 W. Optimized scan parameters for each case study in this research are presented in Table 2.

The other scan parameter to be defined is the exposure time (referred to as integration time or acquisition time in some references). Longer exposure time results in a better signal-to-noise ratio and hence better image quality. Although, too high exposure time can cause the detector to become fully saturated with photons, which generate image artefacts, especially for materials with high absorption tendency.

Another preparation step before scanning is choosing the filter for the X-ray beam, in order to avoid beam hardening. Beam hardening refers to the phenomenon that low-energy beams are more highly absorbed by the object than high-energy beams, causing a shift of the mean energy to higher values as the X-rays pass through the object. The phenomenon of beam hardening makes it difficult to have an accurate quantitative measurement of the attenuation coefficient. To correct the beam-hardening effect, some X-ray suites are equipped with a set of different filters, which helps to absorb the very low-energy photons directly in the filter. On the other hand, filtering decreases the overall intensity, which results in a need for longer exposure time [69]. Filters can be chosen according to transmission value (a pixel-by-pixel division of photon counts of the scanned sample image by the scanned image of air) for different magnifications. Scans with high transmission values do not need any filter. All scans in this paper are in this range.

#### 2.2.3. Contrast Enhancement

As mentioned in Section 1, traditionally, absorption-based X-ray tomographic imaging relies on the density differences between the phases in the sample and has been employed to characterize microstructural evolution. It is clear that this technique has some limitations for studying light density materials (e.g., less than 2 g/cm^3^), particularly in sub-micron resolution. A limitation of high-resolution CT by absorption contrast in the field of polymer composites can be the poor contrast between fibers and epoxy matrix, which often prevents detailed characterization of the material. Hence, this means that it can be difficult to discriminate between elements of the composite, particularly the region between co-aligned neighboring tows [8]. The poor contrast between fiber and resin, as well as a high level of graininess, often hinders the visual characterization of fiber architecture and therefore disrupts volume fraction measurements and fiber orientation, or generation of micro-level finite element mesh for structural analysis.

Over the last decade, several innovative X-ray imaging methods have been established. These methods are based on the phase shift of the X-ray beam passing through a material. This mechanism allows for a substantial increase in the contrast-enhancing interface. Micro-CT phase imaging methods can be classified into (a) interferometric methods, (b) techniques using an analyzer (or diffraction enhanced imaging) and (c) free space propagation-based methods (also called in-line based methods) [70,71].

**Figure 4:**
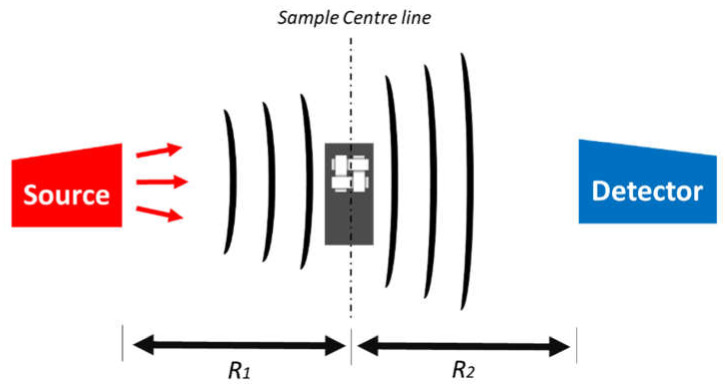
Source, sample, and detector arrangement and spacing during Micro-CT imaging.

**Figure 5:**
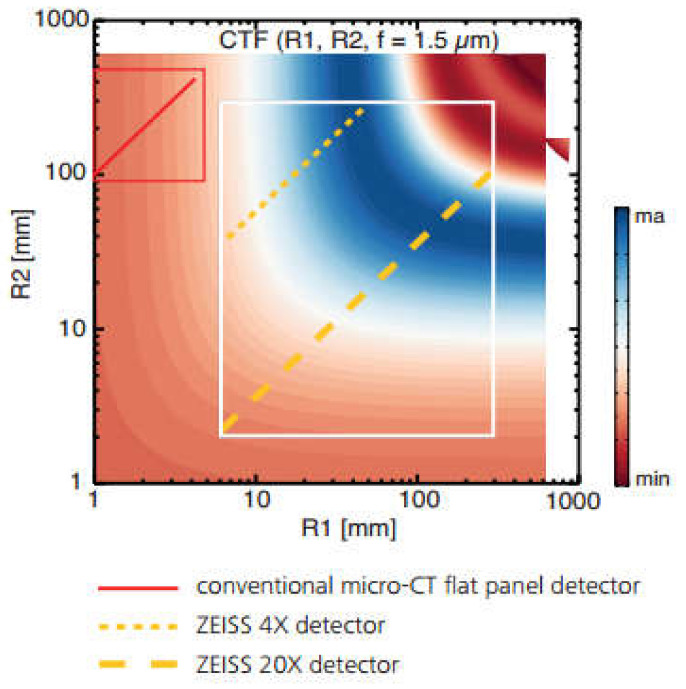
Contrast transfer option (CTF) for a 1.5 μm feature of 40 kV. The white region is the operation window for Micro-CT devices with optimized phase contrast compared to conventional flat-panel Micro-CT (red box) [73].

Propagation-based phase contrast or full phase contrast could be employed as an alternative to tackle this challenge [72]. In this approach, the sample remains in the “near-field” regime and the detector is placed at an optimized distance from the sample. This results in images that display edge enhancement, where the phase of the object can be extracted. Significantly longer scanning time is, on the other hand, a downside for phase-contrast tomography. Xradia/ZEISS Micro and Versa systems are capable of a form of phase contrast, namely, “propagation phase-contrast”. This methodology is based on enhancing the effect of phase fringes that originate at interfaces between similarly attenuating phases. In a practical sense, creating the enhancement is relatively simple; both the X-ray source and the detector need to be moved farther away from the sample. This provides a propagation distance for these interference fringes to develop and become large enough to be resolved by the detector. However, the trade-off is suffering a loss in the throughput of the scan and a subsequently longer scan time of ~96 h needs to be undertaken to absorb a sufficient number of photons for a single scan. The result in the reconstructed data appears as somewhat of an edge enhancement/halo effect. This effect can be produced on a sliding scale, i.e., having the source and detector close to the sample will produce almost a pure absorption dataset (see Section 3.1), while having them increasingly far away will introduce more of the phase effect (R1 and R2 distances in Figure 4). The magnitude of the phase contrast fringe in laboratory CT devices is a function of the energy spectrum of the X-ray beam, the convolution of the source-to-sample distance (R1), and the sample-to-detector distance (R2) (see Figure 4). Flat-panel detectors of conventional Micro-CT systems, which typically have detector pixel sizes on the order of 50–100 μm, are incapable of capturing the majority of phase-contrast information since the detector pixel size is much larger than phase fringe-widths. In contrast, ZEISS X-ray detectors attain pixel sizes as small as a third of a micron (~0.3 μm) and are therefore small enough to capture detailed phase information. The phase-contrast as a function of the X-ray source-sample (R1) and sample-detector (R2) distances is depicted in Figure 5. The contrast transfer function (CTF) demonstrates where image phase contrast is maximized as a function of these two distances. The red line in the upper left represents the operational realm of a typical high-end flat panel-based Micro-CT, while the yellow lines represent the optimum operating range of the medium magnification 4× detector and the high magnification 20× detector on an Xradia Versa suite. The white box in Figure 5 indicates the overall operating range of the instrument [73].

#### 2.2.4. Scanning

The rectangular dry fabric sample was meticulously fixed in the X-ray suite. The sample was centered and aligned with the rotation axis as precisely as possible in a custom grip consisting of a thin foam block (see Figure 3b). The block held the sample in place with minimal contact and pressure, allowing straight but tight placement onto an aluminum mounting rod to mitigate the wobble during the scan. With a magnification of 4×, the scan parameters were optimized for image quality, resulting in isotropic resolutions listed in Table 2. Using Xradia software, 2D radiographs were generated by a 360° rotation of the samples, with a rotation step of 0.144°, for a total of 2500 projections. No filter was used during the scanning process. After finishing the scan, the center of rotation is refined to have an accurate reconstruction [74] by XMReconstructor 8.1 by a filtered back-projection Feldkamp algorithm [75]. The 2D radiographs from the detector are then reconstructed into equally spaced 2D slices via a binning value equal to 1. Additionally, a median filter is used during the reconstruction to decrease the noise in the greyscale images. There are a number of reconstruction algorithms. On top of them is the standard convolution back-projection image reconstruction method. In this study, reconstructed 2D slices were recorded using the tagged image file format (TIFF) and were imported into software Amira-Avizo 9.0 [76] for 3D representation and post-processing analyses.

Similar to the dry fabric case, the prepreg sample was fixed within the Micro-CT suite. As mentioned earlier, the alignment is critical for achieving high accuracy, maximum magnification, and successful reconstruction. Due to the stiffer nature of prepreg, because of the existence of partially cured resin, sample wobble was less likely to occur. Similar to the dry fabric case, 2D X-ray images were acquired for 360° tomographic rotation. There was no physical X-ray filter used in the scanning. The voxel size was chosen to be close to one-third of the carbon fiber diameter (~8 µm), which provides an optimal pixel size for meso-scale segmentation analysis. The scanning parameters are presented in Table 1. Since the prepreg sample consists of liquid and gas phases (pre-impregnated resin and air bubbles, respectively), the optimum scanning parameters were not identical to those applied to the dry fabric sample. The scans were labeled according to the sample material and the scan orientation.

**Figure 6:**
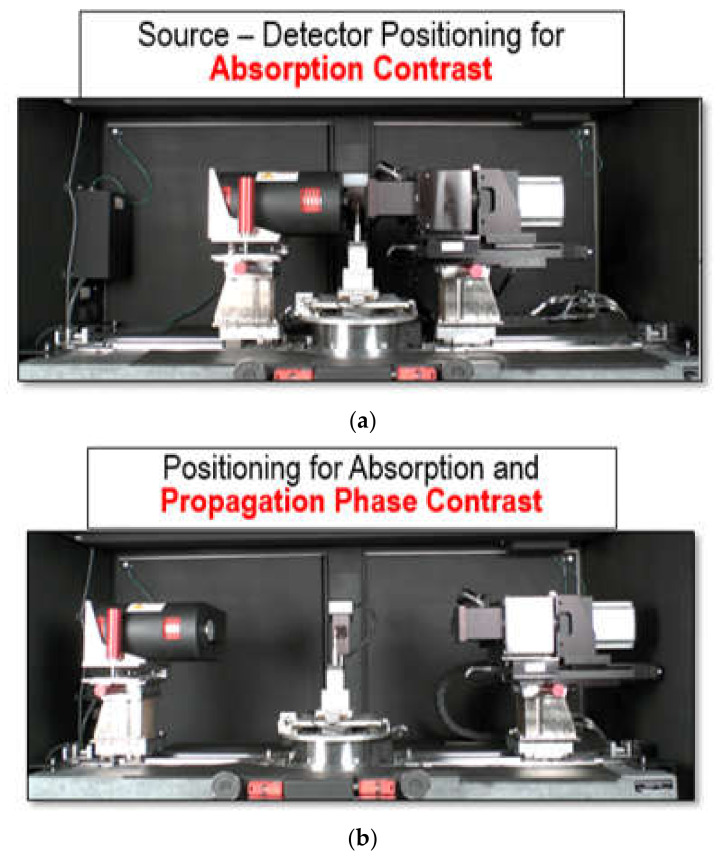
Source–detector positioning for (**a**) absorption contrast; (**b**) phase propagation contrast.

The propagation phase contrast was also conducted on the same prepreg sample. To demonstrate the possibilities of phase-contrast imaging, CT measurements with a rather strong phase contrast were performed. In order to make fair comparisons between the different strategies, in the context of different parameters (Table 2), as many factors as possible were kept constant. When using the same optical lens, it is important to keep the recorded signal approximately constant in each projection, because increasing the number of detected photons will give better counting statistics and thereby affect the signal-to-noise ratio (SNR). However, in order to control the level of phase contrast, the distance between source and sample, $R_{1}$, and sample and detector, $R_{2}$, may vary (Figure 6). Therefore, the exposure time must be changed accordingly to ensure that approximately the same number of photons is acquired in each case. As the number of counts is inversely proportional to the square of the distance between the source and the detector, ${\left(R_{1}+R_{2}\right)}^{2}$, the exposure time required can be calculated from Equation (2) by varying $R_{1}$ and $R_{2}$:

(2) $$t=t^{\mathrm{ref}}.{\left(R_{1}+R_{2}\right)}^{2}/{\left(R^{\mathrm{ref}}+R^{\mathrm{ref}}\right)}^{2}$$

The $R_{1}$-to-$R_{2}$ ratio must be held constant for a given optical magnification to ensure the same geometrical magnification and thereby the same effective pixel size. It is clear from Table 2 that the time per scan varies enormously (by more than a factor of 10) when applying the different scan settings. Table 2 may also influence the operator’s choice of the most appropriate settings. Given that the width of the sample images in the FOV varies with different magnifications, the time to tessellate a number of images to build up an image of the same area varies even more steeply. $R_{1}$ and $R_{2}$ were increased in balance to increase the phase contrast [77], whereas the exposure time, t, was adjusted accordingly to maintain a similar number of counts, as summarized in Table 2. In practice, very large propagation distances ($R_{2}$) should be avoided: first, because excessive phase contrast can blur the edge contrast and, second, because increasing the source to detector distance requires much longer acquisition time.

## 3. Results and Discussion

As previously discussed, here the Micro-CT workflows were tested on two types of samples: a dry woven carbon fabric and a prepreg woven fabric (Table 1). The resolution of the images, voltage, power, and exposure time for each selected imaging case is given in Table 2. In order to provide sufficient architecture for analysis, samples were cut to an approximate width of two tows. Additionally, samples were required to remain completely within the field of view.

### 3.1. Visualization

**Figure 7:**
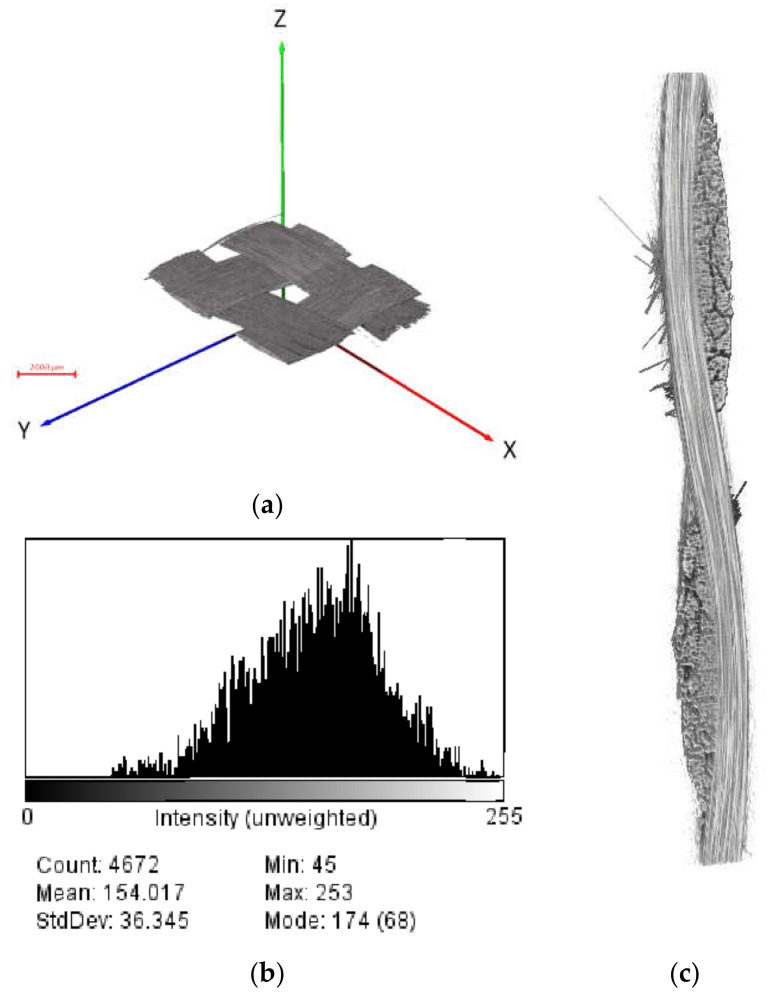
(**a**) Three-dimensional visualization of dry woven fabric in Avizo 9.0; (**b**) Corresponding histogram for the cross-section shown in (**c**). The thresholding range of 125–174 was used to represent carbon fibers; (**c**) a 2D slice from the cross section.

The 1394 TIFF images with 16-bit depth (1988 by 2029 pixels) collected after reconstruction of the dry fabric, totaling 9.45 GB of data, were volume rendered. The reconstructed data were processed and visualized using the Amira Avizo 9.0 software (Waltham, MA, USA). The 3D image was then transformed into the desired coordinate system for further analysis. The recreated 3D volume is presented in Figure 6; note that the rotation axis is parallel to the x-axis. Denser areas appeared brighter in this image. From Figure 7, the arrangement of tows and fiber orientation can be perceived in one RVE of woven fabric. The dry carbon fabric was found to contrast well with air as shown in the same figure. This is the greatest difference considered possible, given the tight arrangement of fibers within the yarn. However, single fibers in the yarn are not completely distinguishable because the distance between individual fibers is less than the effective voxel size.

**Figure 8:**
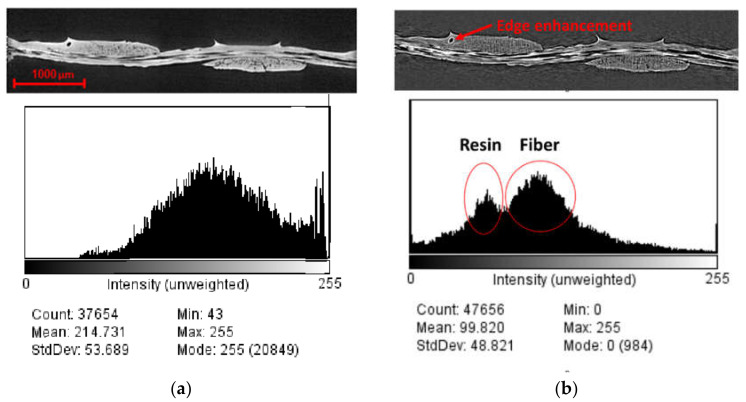
Cross-section of CT-data of a prepreg sample at two different object-detector distances; (**a**) absorption contrast; (**b**) propagation phase contrast. Voxel size was constant at 2.47 μm^3^. Enhanced edge contrast around the void obtained using the phase contrast scan is also highlighted in the figure.

Similar to the dry fabric case, visualizations were obtained after reconstructing the projections for prepreg specimens. The influence of phase-contrast on the resulting CT data and the detectability of details are shown in Figure 8. The slices of the absorption and refraction coefficients exhibit relatively the same features for both CT measurements with the phase-contrast scans provides a better representation of the interfaces between the three phases, although the fibers, resin and voids are easier to resolve in the scan obtained via phase-contrast mode. In particular, edge enhancement between voids and resin was only captured through the latter, as highlighted in Figure 8. The darker regions observed in the samples indicate resin-rich areas between the fiber bundles. The linear attenuation coefficient of the carbon fiber is 0.0457 mm^−1^ at 24.5 keV, which corresponds to the mean energy of the X-ray spectrum used for the measurements. For the epoxy resin, it varies between 0.0450 and 0.0417 mm^−1^ depending on the chemical composition and density. Similarly, the refraction coefficients range is between 4.35 × 10^−7^ and 4.67 × 10^−7^ for the epoxy and amounts to 5.91 × 10^−7^ for the carbon fibers at an energy of 24.5 keV [78]. As a result, the relative difference in the refraction coefficients is much larger than the relative difference of the linear attenuation coefficients, which is confirmed by the higher contrast obtained between resin-rich areas and fiber bundles in the refraction tomogram, compared to the absorption tomogram. However, it is impossible to distinguish between fiber bundles running in different directions since the absorption and refraction coefficients do not depend on the orientation of the fiber bundles. Looking at the Micro-CT results in Figure 8b, three different levels of grey can be distinguished in the sample. As shown in the histograms, the darker tone corresponds to the epoxy resin between bundles while the impregnated bundles appear in lighter greys. The cross-sections were extracted from the same plane within the sample. Increased object-detector distance results in an increase in the phase contrast. The visible phases are voids, polymer resin, carbon fibers perpendicular to the section plane and carbon fibers parallel to the section plane. Voids in the matrix between the fiber bundles, as well as small voids within the fiber bundles, were visible in the phase contrast scan. It should be noted that these voids are the unfilled spaces between the fibers and resin in the uncured prepreg investigated and originate from the prepreg fabrication process. For the absorption contrast case in Figure 8a, fiber and resin cannot be distinguished as also suggested by the histogram, whereas two peaks can be distinguished for fiber and resin in Figure 8b for the phase contrast scan.

**Figure 9:**
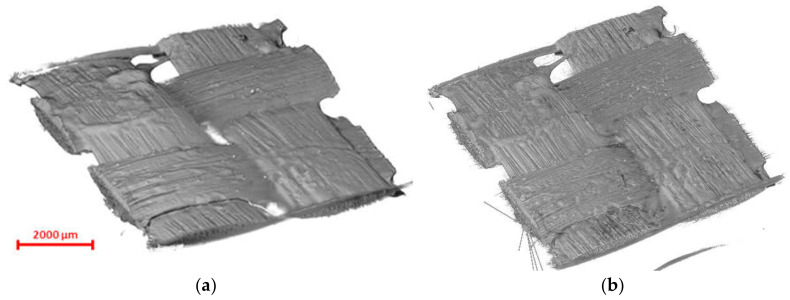
Three-dimensional rendering of the prepreg sample (**a**) absorption contrast and (**b**) propagation phase contrast. The thresholding range of 133–182 was used to represent carbon fibers after normalizing the histogram for both cases.

**Figure 10:**
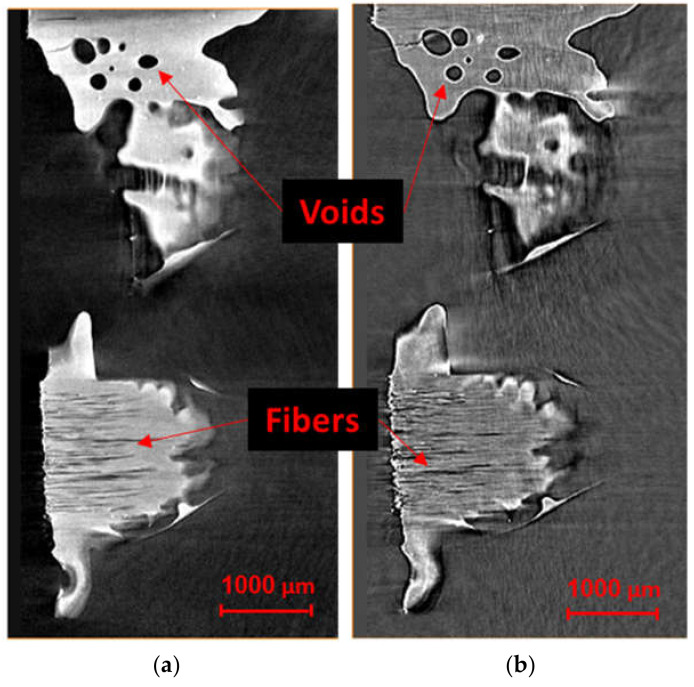
Top-view of fiber directions and air bubbles in (**a**) absorption contrast and (**b**) propagation phase-contrast scans.

To attain a complete picture of the tow architecture and surface resin distribution, a spectrum of grayscale shades from each cross-sectional image was selected for the three-dimensional visualization. The upper and lower limits of this spectrum were used as threshold values in converting the cross-sectional images into binary images. As shown in Figure 9, small grooves appear on the surface of the yarns and are aligned in the fiber direction. This obscures the actual resin-rich areas on the surface and in the through-thickness sections, which should be analyzed to confirm whether they are part of the voids and not artefacts. This may be the result of small dimensional changes in the tow surface, the magnitude of which has been exaggerated by the Micro-CT scanning process. The phase-contrast scan showed to be more effective in detecting the edges of the voids and boundaries of contacting tows (see Figure 10), yet the absorption case was sufficient for enhancing contrast for tow visualization.

All reconstructions did not require consideration of artefacts in the CT process, such as ring and beam hardening, after reviewing the output images. These types of defects occur when the FOV is smaller than the sample size, which can functionally distort the information about the sample, causing improper identification of zone densities and geometries [79]. Beam hardening was the main artefact observed at the center of the sample, albeit with a mild presence, which needed to be addressed in post-processing. Beam hardening occurs when the lower frequencies of the polychromatic photons are attenuated more than higher frequency photons. This leads to the image artefacts of “streaking” and “cupping”, where the former is the result of the attenuation occurring at different rates throughout the sample, and the latter describes a peripherally dense appearance in images due to lower-density materials further along the beamline not scattering the remaining high-energy photons. In this case study, beam hardening occurred due to the difference in density between additives within the polymer resin and the surrounding composite, but its magnitude was not significant enough to warrant re-running the scan procedure to obtain new images. Hardening artefacts at the corners of the samples were also less of a problem since the architectures of interest were generally located at the center of the samples. Ring artefacts, which did not occur in the acquisition of these samples, are caused by elements in the detector array that are either faulty or miscalibrated, leading to “rings” of improper contrast around the axis of sample rotation.

Overall, phase contrast provides a simple and general method for improving the contrast in radiographs from weakly absorbing samples and requires access to only conventional tomography devices/sources. Yet, longer exposure times can be deemed as the main drawback, making this technique less efficient, as it is more time consuming. This can only be justified if detailed micro-mechanical analysis needs to be undertaken. The experimental configuration for obtaining phase-contrast scans may need to be optimized in order to operate under the least scanning time, without substantial loss in contrast. High-resolution cone-beam CT allows for a detailed inspection of the microstructure of CFRP samples, in particular of the porosity and fiber positions. The phase-contrast gained by increasing the object-to-detector distance increases the contrast at interfaces and allows single filaments to be resolved. Nevertheless, the size of the region of interest (ROI) in this method, due to the large magnification factors, can heavily limit the practicality of its use.

### 3.2. Post-Processing and Analysis

#### 3.2.1. Filtering and Segmentation

**Figure 11:**
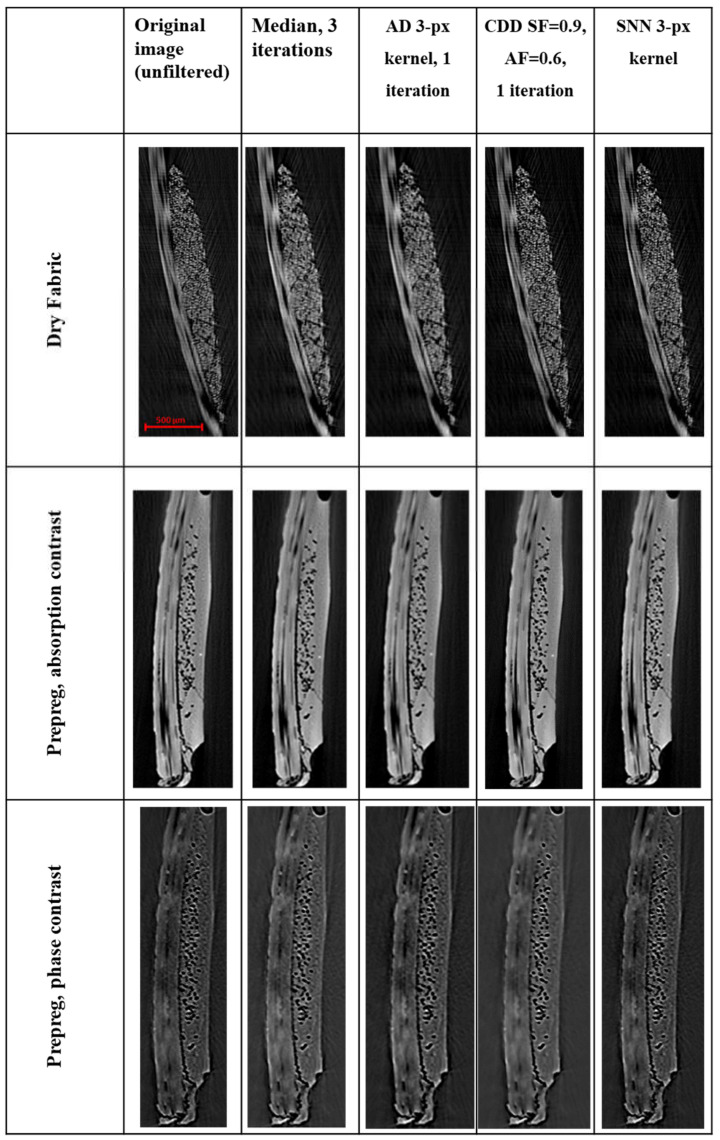
Selected cross-sections of scanned samples of dry fabric, prepreg with absorption contrast and prepreg with phase-contrast scanning strategies. A zoomed-in view of the composite cross-section has been provided, for ease of comparison with respect to image details. Each image has had four filters applied for comparison: Median (3 iterations), Anisotropic Diffusion (3-pixel kernel, 1 iteration), Curvature-Driven Diffusion (SF = 0.9, AF = 0.6, 1 iteration) and Symmetric Nearest Neighbor (3-pixel kernel).

After obtaining sectioned images of the scanned fabric samples, different image filtering strategies were tested and compared (see also the Appendix A for more in-depth understanding of these strategies via mock-up trial and errors). The hyperparameters associated with each filtering algorithm were selected (Figure 11), as applied to the dry fabric, prepreg with absorption contrast and prepreg with phase-contrast scanning strategies. As can be seen from the images presented in Figure 11, each filtering algorithm provides different results in image quality. In particular, it was noted that there is a significant presence of undesirable features in the original, unfiltered images. In the case of the dry fabric, this is particularly noticeable in the form of artefacts from the Micro-CT around the periphery of the sample boundary, as well as noise within the yarn cross-sections. With respect to the prepreg samples, there is significant heterogeneity of the greyscale values in the resin regions, as a result of noise.

With the application of the four filters to these images, it was seen that there are visible changes of the features within the image. In particular, the application of the Median and Anisotropic Diffusion filters has yielded a much blurrier image in the intra-tow region of the fabric. As it may be important that individual filament positions be trackable between cross-sections, this blurriness can reduce the ability to capture such a feature and is highly undesirable. This is more pronounced in the dry fabric image but is also a significant concern in the prepreg images, where the boundaries of the intra-yarn voids are similarly blurred. Similarly, the Symmetric Nearest Neighbor (SNN) filter introduces blurriness, although within a smaller neighborhood of pixels compared to the first two filters. This can be intuitively described as producing a “serrated” edge effect, where once smooth edges now have a coarse texture. As the kernel size is increased, this noise is only exacerbated and finer features, such as the intra-yarn filaments, begin to exhibit a blurring more akin to the Median and Anisotropic Diffusion filters. The Curvature-Driven Diffusion filter yielded the best results in this case study, especially due to its ability to both provide smoothing of noise, as well as preserve edges, particularly of fine details. Having empirically determined that the use of a Curvature-Driven Diffusion (CDD) filter is visually optimal for application in Micro-CT scan results of woven fabrics, both dry and prepreg, the filter was applied to the entire stack of images for further processing.

After enhancing the quality of an image by the application of post-processing filters, segmentation techniques were employed to partition the image into four different yarns which construct the unit cell (see Figure 9). This is performed to make it easier to distinguish between different phases of the specimen. Segmentation highly depends on the quality of the image and the method by which the details are reconstructed. The study [80] divided segmentation methods into the general categories of classical methods, pattern recognition-based methods, deformable models, wavelets-based methods, atlas-based methods, and knowledge-based techniques.

For woven fabric composites, different segmentation tools can be used depending on the desired outcome of the process. One of the applications of segmentation in the fabrics is separating yarns in the dry fabric with the purpose of, e.g., measuring the degree of misalignment between yarns. If there is a distinctive difference between fibers’ density and the background space, thresholding is the fastest and easiest way to separate yarns of the fabric [81]. VoxTex software, developed at KU Leuven, is an image processing software widely employed for detecting warp and weft tows of woven fabrics using the eigenvalues of the 2D structure tensor [82,83]. In this statistical-based method, warp and weft tows are separated by defining the degree of anisotropy. Segmentation results are then used to measure fiber volume fractions inside the yarns, characterize in-plane misalignment of yarns, and mesh the structure in software such as Dassault Systèmes^®^ Abaqus. In another work, Naouar et al. [83] utilized Texture Analysis for the image-based segmentation of a 3D E-glass woven fabric. This algorithm works based on the calculation of the Gray Level Co-occurrence (GLC) matrix. Textural features such as contrast, correlation, energy, and homogeneity can all be extracted from the GLC matrix. Following this, warp and weft yarns are separated by a threshold using homogeneity [83].

**Figure 12:**
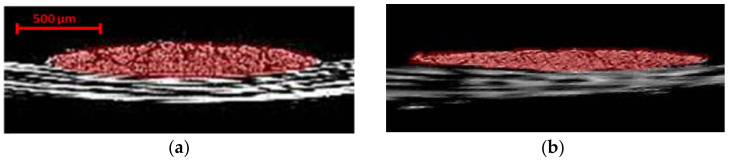
(**a**) A good estimation of a yarn section by ellipse shape; (**b**) a yarn section that cannot be estimated by ellipse shape.

**Figure 13:**
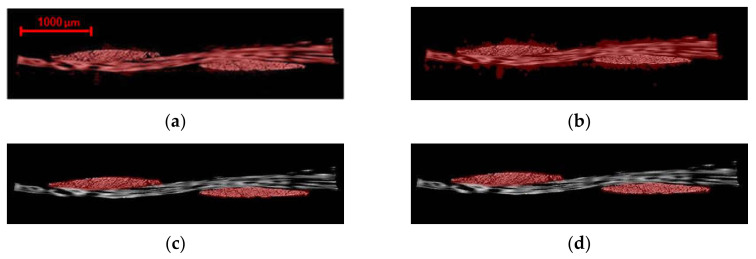
Segmentation steps: (**a**) thresholding; (**b**) growing; (**c**) trimming by brush tool; (**d**) after smoothing and filling.

In this research, Avizo 9.0 software was used for segmentation. Segmentation tools within the software to label pixels of the image can easily apply a variety of segmentation methods. Lasso and brush were used to split the material into selected regions manually. For segmentation in Avizo, it is necessary to resample the image data to reduce computation time and improve rendering performance. To this end, the resolution of the image was decreased by changing the pixel size from 2.88 µm to 9.5 µm. Adopting the strategy taken by Yousaf et al. [84], yarn cross-sections are manually selected in 18 of 530 slides and the meso-scale yarn shape was generated via interpolation. Cross-sections can be selected manually by the brush or lasso tools or can be estimated by the closest ellipse shape as performed in [85]. This approach is easier to use, but by looking at different image slices, it can be inferred that not all the yarn cross-sections are reasonably estimated by the ellipse shape shown in Figure 12. Thresholding can select all the pixels between the two defined intensities. However, the empty space between fibers is not selected, whereas the yarns in the other direction are selected. To overcome these issues, the grow tool in Avizo was used to fill small holes inside the selected region, then excessive regions were removed. Following this process, smoothing was utilized to help homogenize the selected region. Segmentation steps are shown in Figure 13 for slice 100 in the XZ direction. After marking the yarn cross-section for all 18 slices, the remaining slices were marked by interpolation and the completely marked yarn were assigned to a new material label.

#### 3.2.2. Geometry Extraction and Meshing

A finite element (FE) mesh can be generated from the segmentation step, in turn, generated from the Micro-CT images. This allows the geometric model to account for real-world variabilities such as tow waviness, voids and thickness variations within the representative volume element (RVE), compared to idealized geometries generated by the software; although some degradation in the resolution is inevitable due to the dependency of the generated FE mesh to the segmentation operation. In meso-scale textile composite modeling, the yarn is considered as a 3D domain. To apply various constitutive models to yarns inside a 3D woven fabric unit cell, a detailed and precise description of both the fabric topology and yarn geometry is required. Following the segmentation process, a multi-step algorithm was used in this study to convert the resulting virtual-fabric data to a 3D mesh, to be able to be used in finite element software for numerical analysis. Care should be taken in executing the segmentation process, particularly in the areas where yarns have contact interaction, such as cross-over points. This was done during the segmentation step by introducing labels for every single yarn at contact zones to prevent overlap in the selection of the fibers from adjacent yarns. The change in cross-section and hence the length of the yarn in the final mesh is correctly captured through the interlacing and contact of the yarns, only if the edges are not over/underestimated. In generating the as-woven geometry, the surface of the yarns must be extracted first, then meshed as solid entities afterwards. The generation process involves extracting iso-color surfaces which follow the segmentation into the indicated four regions from the segmentation processing output, followed by simplifying the surface. The uniformly distributed points from the former are defined as nodes and nodal connectivity, which is prescribed to generate a structure of hexahedral or triangular elements, accurately representing the as-woven geometry.

**Figure 14:**
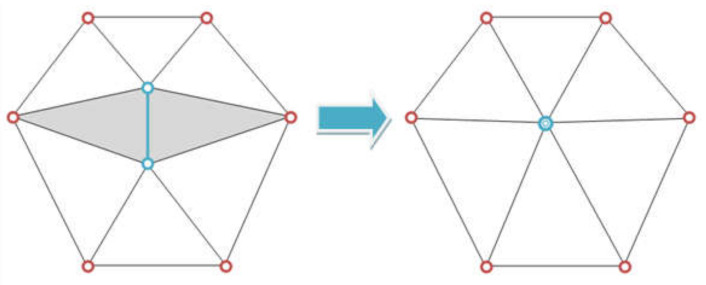
Illustration of edge collapsing technique, where the blue edge is collapsed into a single point. The shaded triangles degenerate and are removed during the contraction.

Amira Avizo 9.0 was used to compute a triangular approximation of the interfaces between different regions via the marching cubes algorithm [86]. Smoothing is essential [87], because the segmented images often consist of cuboid elements (voxels) that without correction, would produce staircase-like surfaces. To smooth out region boundaries, surface simplification is done using an edge-collapsing algorithm, where edges of the original surface are successively reduced to points, as shown in Figure 14. The shape of the original surface is preserved by minimizing a chosen error criterion. Special care should be taken to prevent the triangles of the simplified surface from intersecting each other. Meshes of the yarn can be generated with hexahedral, quadrilateral, triangular, and tetrahedral elements. Nonetheless, neither of these element shapes is completely acceptable. Generally, the hexahedral elements are numerically more efficient, and they are well adapted to describe the yarn in the fiber direction [88]. However, it is problematic to mesh the transverse section of the yarn in a suitable manner [82]. Most of the composite yarns have a lenticular shape and, therefore, both extremities are difficult to mesh. On the other hand, the tetrahedral elements can mesh any volume and they efficiently describe the transverse section of the yarn. However, a mesh-based on tetrahedrons needs a large number of elements to be adequately precise.

#### 3.2.3. Simulation

**Figure 15:**
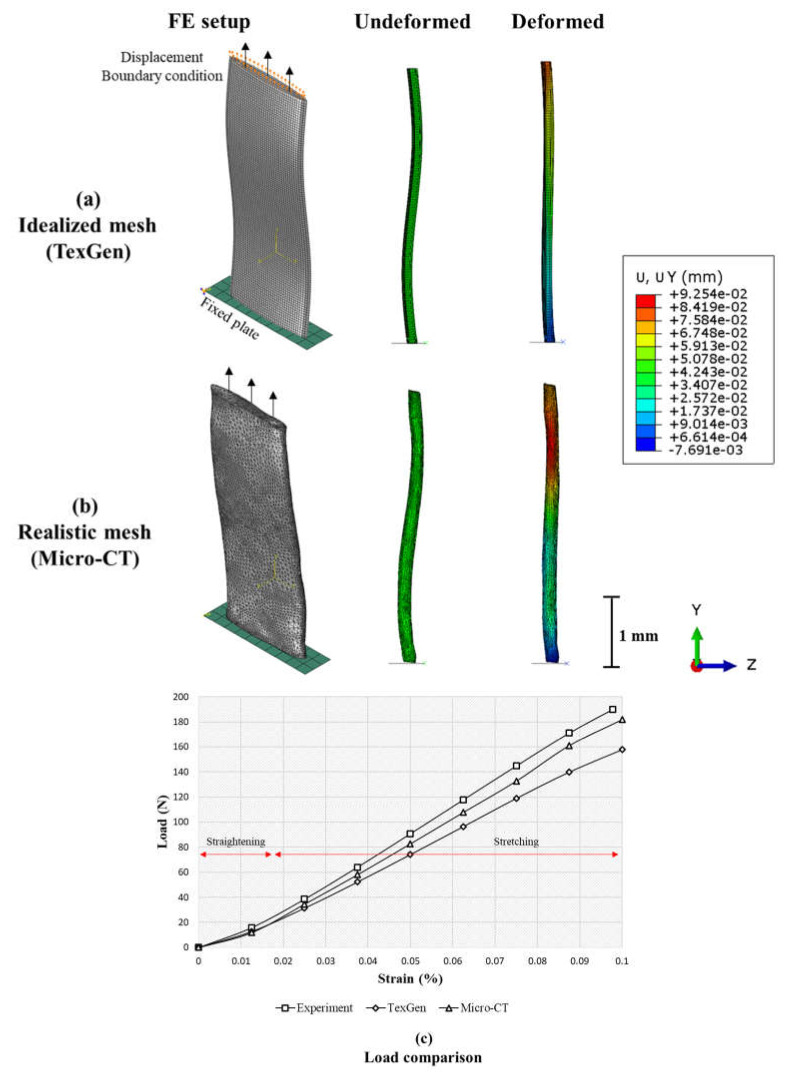
Uniaxial loading Finite element (FE) simulations: (**a**) Idealized mesh generated via Texgen software (**b**); Generated FE mesh using triangular elements for the dry woven fabric case accounting for the structural variations. (**c**) Comparison between the load response up to 0.1% strain for all three cases. The onset of stretching is also shown in the figure.

To illustrate the modeling workflow, a linear quasi-static uniaxial finite element (FE) analysis was next performed to compare the structural response of an idealized yarn geometry generated via TexGen [89] software with the realistic geometry obtained from the present Micro-CT scans. The simplified segmented data were exported from Amira Avizo 9.0 to the Geomagic Wrap software as DXF (Drawing Exchange Format) surfaces to smoothen and partition the surfaces. The geometry was then imported to Abaqus FE software in STP format to perform the numerical analysis. As shown in Figure 15, the responses of the two yarns were investigated under tension by fixing the yarns’ ends to a rigid plate and applying displacement equivalent to 0.1% strain to the other. The FE analysis was conducted with the material properties of E_1_ = 230 GPa, E_2_ = E_3_ = 9.8 GPa, G_12_ = G_13_ = G_23_ = 2.8 GPa, ν = 0.3 for the carbon fiber yarn; where index 1 refers to the fiber-direction and 2 (in plane) and 3 (through-the-thickness) are perpendicular to 1; E refers to the elastic modulus, G is the shear modulus, and ν is the Passion’s ratio. Local coordinate systems were used to align the stiffness direction of the fiber bundles into their respective material orientations. The deformed shapes along with response curves for each scenario are shown in Figure 15a,b, respectively. The idealized model led to an underestimation of stiffness compared to the experiment due to the overestimating of actual yarn waviness, whereas the simulated geometry models produced results closer to the experiment. As shown in Figure 15c, load curves are composed of two parts, namely, the straightening (suppression of yarn undulation), and fiber stretching. The overall stiffness of the yarn was found to be closer to the experiment due to the more realistic thickness profile in the cross-section of the micro-CT model. This explains the importance of accounting for geometrical variability in the numerical analysis of woven fabrics. Further, the high degree of similarity between numerical and experimental results indicate that the fabric morphology has been accurately captured from the micro-CT process. Alternative methods, such as direct volumetric analyses comparing the true volume of the fabric and the rendered mesh, can also be used to validate the image-capture and mesh-generation workflows.

## 4. Conclusions and Future Prospect

The growing interest in micro-computed tomography (Micro-CT), as one of the most resourceful tools for high-resolution 3D imaging and non-destructive analysis of materials and manufacturing, reflects the need for its further standardization/customization for different applications; otherwise trial and errors can often become very costly and time-consuming to arrive at the appropriate level of pre- and post-scan parameters. After a review of the state of the art in the file on the woven composites, this work conducted a comparative case study towards 3D Micro-CT imaging, visualization, and numerical reconstruction of low X-ray absorptive (difficult to scan) carbon woven fabrics at the constituent-level length scale, using aerospace-grade dry and prepreg samples. Different filtering and segmentation algorithms were discussed to convert the obtained tomographic data into meaningful numerical representations. Finally, the segmentation data were converted into a solid model representation of the material, which was then meshed to be used within finite element (FE) models. A simple uniaxial tension numerical analysis on a yean was performed to assess the performance of Micro-CT-based mesh. The possibilities, advantages and restrictions regarding obtaining sufficient contrast, an examination of thin samples, sample size/resolution issues, and quantification of defects were elaborated on.

The combined imaging, segmentation, filtering, and meshing procedures described herein may provide a preliminary workflow towards further development of best practices on virtual testing of CFRPs and its applications. In particular, by obtaining high fidelity numerical models of carbon woven composite fabrics via actual images of the material microstructures, new advances can be made towards an in-depth understanding of the constitutive materials behavior as well as micro and meso-scale deformation modes during the complex forming process of these materials, such as intra-yarn shear and wrinkling behavior [90]. The quality of such analyses, however, will be highly sensitive to the quality of the scans themselves. Despite the promising results shown thus far on the Micro-CT investigation of woven composites by different researchers, a number of important technical challenges remain to address. For instance, in lab-scale tests similar to the present case study, a relatively small number of unit cells of the woven structures can be scanned. For practical applications, it is necessary to acquire such data from larger, macro-scale components with off-axis fiber stacking sequences, over limited scan angles (e.g., <180°). As a result, the present segmentation algorithm must be extended to enable efficient processing of larger volumes with lower resolution, noisier, and perhaps incomplete data. The results obtained through phase-contrast CT scanning of woven prepreg using a lab-scale CT device hardly justifies the long acquisition time needed to perform such a task and hence more efficient avenues such as synchrotron-based micro-computed tomography might be a viable option for microscale characterization of smaller intra-bundle voids and individual filaments, given the higher resolution and significantly shorter scan times (minutes vs. hours of micro-CT), allowing for capturing an extended area on the specimens at a reasonable time and expense.

## Figures and Tables

**Table 1:** Specifications of the selected dry and prepreg fabrics in this study.

Manufacturer	Cytec
Architecture	3K–Plain Weave
Area density (g/m^2^)	2722
Sett (yarns/cm)	3.5
Filament diameter (µm)	7.93
Nominal thickness (mm) (dry fabric)	0.295
Nominal thickness (mm) (prepreg)	0.33
Resin type (prepreg)	CYCOM 970 Epoxy Resin

**Table 2:** Scan parameters used for three imaging cases selected.

Case	Voxel Size (µm^3^)	Voltage (kV)	Power	Exposure Time (s)	Sample to Detector Distance (mm)	Sample to Source Distance (mm)
Dry Fabric	2.88	40	10	20	8	47
Prepreg (absorption contrast)	2.47	40	15	5	12	32
Prepreg (phase-contrast)	2.47	40	20	210	300	500

**Figure A1:**
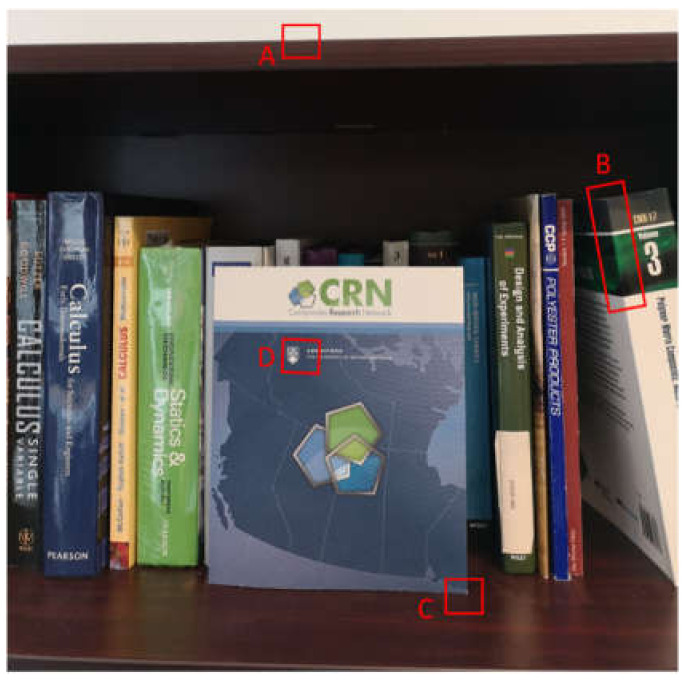
A mockup photograph used first to analyze the effects of different image filtering algorithms on an ultra-high-quality base image. The image was selected due to its multiple features that are relevant to the conducted cases of fabric Micro-CT images. Namely, these included: (A) high contrast regions with high gradients along the axes of pixel distribution (x, y directions), (B) similar high contrast regions at off-axis orientations, (C) curved edges with parallel texture features in the foreground and depth-of-blur applied to the background, as well as (D) high-aspect-ratio linear features in low-contrast regions. Having multiple regions in various combinations allowed for the independent appraisal of global and local effects of the filtering methods, to better choose which would be suitable for the case study at hand.

**Figure A2:**
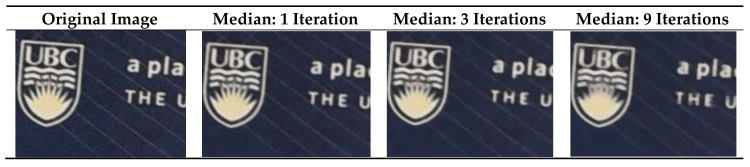
Comparison of the results between the original image from region D in Figure A1 to cases where the Median filter was applied at one, three and nine iterations.

**Figure A3:**
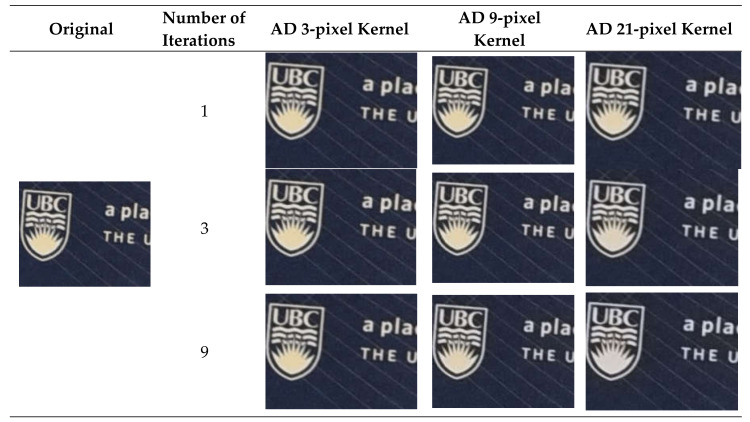
Comparison of the results between the original image from region D in Figure A1 to cases where the Anisotropic Diffusion filter was applied at one, three and nine iterations (rows), in addition to varying kernel sizes of three, nine and 21 pixels (columns).

**Figure A4:**
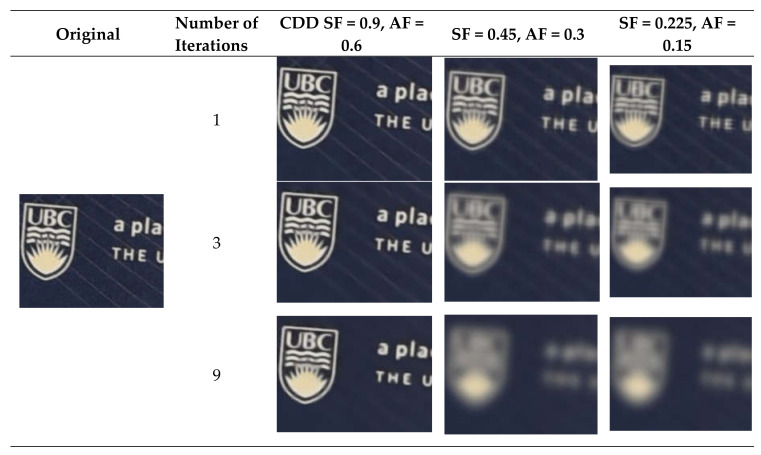
Comparison of the results between the original image from region D in Figure A1 to cases where the Curvature-Driven Diffusion filter was applied at one, three and nine iterations (rows), in addition to varying sharpness factors (SF) and aspect factors (AF) (columns).

**Figure A5:**
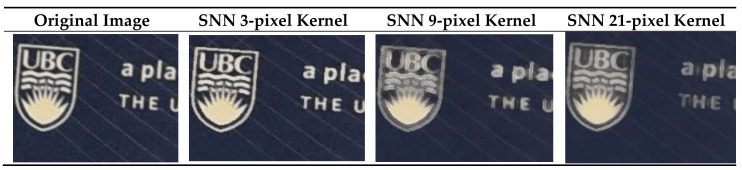
Comparison of the results between the original image from region D in Figure A1 to cases where the Symmetric Nearest Neighbor (SNN) filter was applied with neighborhood sizes of 3, 9, and 21-pixel spans.

## Appendix A. Understanding the Effect of Filtering and the Necessity for Trial and Errors

As addressed in Section 1.1, obtaining high-quality x-ray radiographs and reconstructed tomographic volumes of woven fabric composite materials is often uncertain and subject to the introduction of uncontrollable and undesirable information; specifically, noise [64]. Therefore, the application of filtering, to remove this noise as an unwanted component of the image-based signal [91], is an integral step in micro-CT practice, which in turn can maximize the quality of the scan results as well as the subsequent post-processing and numerical analysis. In order to better understand the effect of different filters, a mock-up test has been demonstrated in this appendix.

Filtering in the context of multiple feature objectives (such as CFRPs in the presence of material, fiber, and interfaces) is a non-trivial task and requires experience and often trial and error. Figure A1 shows the selected mock-up, which is an ordinary photograph taken in a setting with a multitude of features to be analyzed when applying filters. Filters for the purpose of sharpening and smoothing are the focus of most post-processing Micro-CT studies. Here, four filtering algorithms were selected and compared; median, antialiasing, curvature-driven diffusion (CDD), and symmetric nearest neighbor (SNN). These filters were chosen mainly due to their prevalence in similar Micro-CT studies in the literature associated with scanning low density and low contrast materials [92], as well as their availability in the Avizo software used for the reconstruction of tomographs. To appraise the quality of the filters, they were applied to different regions of the image in Figure A1, in different configurations (i.e., different constants selected for each algorithm). Given the relevance of Region D highlighted in the image, with respect to the high aspect ratio and low contrast gradients of woven composite fabrics, the results from this region are presented in the comparisons to follow.

The non-linear Median filter is one of the most commonly applied filters, in part due to its computational simplicity [93]. The filter considers a square neighborhood of pixels around a central pixel, the size of which is specified by the user, across which an average calculation of pixel values (in the case of this study, 8-bit greyscale values between 0 and 255) is made. This average value then becomes the value for the central pixel. This calculation is given by Equation (2). The purpose of this algorithm is for smoothing, typically by removing overly bright or dark spots in a local region that introduces noise to other local features of interest. In the context of composite materials, defects such as metal, sand, dust or other potentially high-density occlusions can introduce such noise, which median filters can reduce. In the Avizo software used, the size of the Median filter uses a 3 by 3-pixel sampling window and users define the number of iterations applied to the image. Figure A2 illustrates the effect of multiple iterations of this filter on region D of the image in Figure A1. It can be seen from Figure A2 that as the number of iterations increases, while the effect of bright and dark spot artifacts may be diminished, information pertaining to small features, such as the parallel lines and text (analogous to filaments in a yarn), is progressively lost.

(A1) $$x_{i,j}=round\left(\frac{\sum_{n}^{i=1}x_{i}\sum_{n}^{j=1}x_{j}}{n}\right)$$

Anisotropic diffusion (AD) is another common form of filtering, for the purpose of sharpening edges [92]. This is deemed important in this case study, as relatively low resolution (2000 × 2000) of the tomographic images, as well as the low greyscale depth (8-bit), can result in “jagged” boundaries between features such as filaments and surrounding materials. This reduces the clarity of images and can lead to further noise in subsequent post-processing. Equation (A2) shows the mathematical approach for applying AD, through the application of local gradients of pixel values to determine the level of smoothing requires, where div is the divergence operator, $c\left(x,y,t\right)$ is the diffusion coefficient, ∇ is the gradient operator and Δ is the Laplacian operator. Figure A3 demonstrates the results obtained from the application of this filter on region D of the image shown in Figure A1, using the two hyperparameters available in the Avizo software; kernel size and the number of iterations applied. As can be seen, there is little effect from this filter on this region, as the high resolution and bit-depth mean that edges were very defined and high-quality to begin with. There is, however, a noticeable change where regions of similar light and dark tones are accentuated further to more white and black shades, due to the divergence operator in the function.

(A2) $$\frac{\partial I}{\partial t}=div\left(c\left(x,y,t\right)\nabla I\right)=\nabla c.\nabla I+c\left(x,y,t\right)\Delta I$$

Curvature-driven diffusion (CDD) filtering is an advanced technique of filtering that seeks to provide both sharpening and smoothing attributes, by accommodating local geometrical features. CDD is a total variation and non-linear diffusion method and has been shown to be effective in preserving thin structures [76]. The equation is the formulation that drives the filtering algorithm, where φ is a monotonically increasing function, G is a diffusivity function and $\nabla^{2}u\;\left(x,y\right)$ is the Laplacian of the image function. In the CDD filter used, the embedded diffusivity function has two hyperparameters that are user-defined; the sharpening factor (SF) and aspect factor (AF), which are both normalized between 0 and 1. The former provides gradient-based control, whereas the latter provides control over the size of the pixel neighborhood of the filter. AF is of particular importance with respect to preserving thin geometric structures [94], such as filaments in a fabric yarn. Attention must be paid towards the absolute number of pixels that define these structures. Figure A4 illustrates the results of applying the CDD algorithm using three different levels of SF, AF and number of iterations, respectively. The results indicate that increasing the number of iterations yields results similar to that of Median filters, washing out the presence of thin and high-contrast geometries. By decreasing both SF and AF, the algorithm tends to favor smoothing as opposed to sharpening, especially for the fine details present in the sample image. With even larger features losing significant information at mid-range choices of SF and AF, it was deemed that for the fine structure of fabric filaments, only high values should be used for filtering.

(A3) $$\frac{\delta u}{\delta t}=-\nabla^{2}\left[\varphi \left(G\right)\nabla^{2}u\left(x,y\right)\right]$$

Symmetric nearest neighbor (SNN) filters are considered to be one of the most powerful and robust filtering algorithms for a variety of applications [95]. The algorithm operates by comparing opposite pairs of pixels in a local neighborhood, selecting those that are closest in value to the central input pixel and returns the mean value of those selected pixels. This method poses various advantages over other filters, such as avoiding excessive smoothing over edges. The user-defined input for this method is the size of the neighborhood over which the algorithm is applied (kernel size). Figure A5 shows the results for different kernel sizes applied to the previously identified region of interest in the sample image. It has been found that the quality of results is highly coupled to the size of the features of interest. For example, for a 3-pixel kernel, the results are considered to be excellent, providing superior edge preservation. However, when the kernel size is increased, artifacts begin to develop, due to a greater number of pixels being incorporated into the algorithm and in turn muddying bright and dark regions. In the 9-pixel kernel case, pixels are artificially lightened in enclosed sections within alphabetic characters (such as “a” and “p”), indicative of approximately 9-pixel spans. Similarly, when the kernel is increased to 21 pixels, in this image, that span causes spaces between alphabetic characters to lighten. Both of these outcomes are undesirable and highlight the need for users to be cognizant of feature sizes when applying these filters.

As demonstrated above, there is a wide variety of image filters that can be selected for use in the sharpening and smoothing of multi-feature images. However, the optimal filter(s), as well as the associated hyper parameters, must be chosen carefully with the specific features that the user is seeking to capture. In the sample image used above, the primary region D of interest contained features analogous to that of high aspect ratio filaments in composites (see the thin parallel lines, e.g., in Figure A5). Typically, sharpening filters such as Anisotropic Diffusion did not significantly affect the quality of the already ultra-high-quality mock-up photograph, whereas smoothing filters such as the Median filter led to a loss in quality when the hyper parameters covering large regions of pixels were selected. Hybrid filters, especially the Symmetric Nearest Neighbor filter with low kernel size, showed the best results.
